# Biochemical Analysis of Urine Samples from Diabetic and Hypertensive Patients without Renal Dysfunction Using Spectrophotometry and Raman Spectroscopy Techniques Aiming Classification and Diagnosis

**DOI:** 10.3390/bioengineering9100500

**Published:** 2022-09-24

**Authors:** Elzo Everton de Sousa Vieira, Landulfo Silveira, Henrique Cunha Carvalho, Jeyse Aliana Martins Bispo, Fernanda Barrinha Fernandes, Adriana Barrinha Fernandes

**Affiliations:** 1 Biomedical Engineering Institute, Universidade Anhembi Morumbi (UAM), Rua Casa do Ator, 275, São Paulo 04546-001, Brazil; 2 Centro de Inovação, Tecnologia e Educação (CITÉ), Parque Tecnológico de São José dos Campos, Estrada Dr. Altino Bondensan, 500, São José dos Campos 12247-016, Brazil; 3 Department of Electronic Engineering, Universidade Tecnológica Federal do Paraná (UTFPR), Via Marginal Rosalina Maria dos Santos, 1233, Campo Mourão 87301-006, Brazil; 4 Center for Biological and Health Sciences, Universidade Presbiteriana Mackenzie (UPM), Rua da Consolação, 930, São Paulo 01302-907, Brazil

**Keywords:** Raman spectroscopy, urinalysis, diagnosis, diabetes, hypertension, biochemical analysis, quantification, discriminant analysis

## Abstract

The purpose of this study was to perform a comparative biochemical analysis between conventional spectrophotometry and Raman spectroscopy, techniques used for diagnoses, on the urine of healthy (CT) and diabetic and hypertensive patients (DM&HBP). Urine from 40 subjects (20 in the CT group and 20 in the DM&HBP group) was examined in a dispersive Raman spectrometer (an 830 nm excitation and a 350 mW power). The mean Raman spectra between both groups showed a significant difference in peaks of glucose; exploratory analysis by principal component analysis (PCA) identified spectral differences between the groups, with higher peaks of glucose and proteins in the DM&HBP group. A partial least squares (PLS) regression model estimated by the Raman data indicated the concentrations of urea, creatinine, glucose, phosphate, and total protein; creatinine and glucose were the biomarkers that presented the best correlation coefficient (*r*) between the two techniques analyzed (*r* = 0.68 and *r* = 0.98, respectively), both with eight latent variables (LVs) and a root mean square error of cross-validation (RMSecv) of 3.6 and 5.1 mmol/L (41 and 92 mg/dL), respectively. Discriminant analysis (PLS-DA) using the entire Raman spectra was able to differentiate the samples of the groups in the study, with a higher accuracy (81.5%) compared to the linear discriminant analysis (LDA) models using the concentration values of the spectrometric analysis (60.0%) and the concentrations predicted by the PLS regression (69.8%). Results indicated that spectral models based on PLS applied to Raman spectra may be used to distinguish subjects with diabetes and blood hypertension from healthy ones in urinalysis aimed at population screening.

## 1. Introduction

Urine is a body fluid consisting of approximately 95% water and approximately 5% of urea, creatinine, uric acid, phosphate, and other compounds [1]. It is widely used for the diagnosis of a range of health conditions in an individual or group. Urea and creatinine are the major components of human urine, and changes in urea concentration in blood, serum, or urine may indicate pathologies such as renal failure, hyperpyrexia, leukemia, diarrheal diseases, diabetes, and hyperthyroidism [2]. Creatinine is the end product of muscle metabolism, commonly used to test renal filtration function [3]. The glomerular filtration rate (GFR) is used in clinical practice as a standard measure for evaluating renal function and is an important indicator for the detection, assessment, and treatment of chronic kidney disease [4]. According to Montero et al. [5], proteinuria is one of the most common clinical manifestations of diabetic nephropathy and is closely related to the transdifferentiation of mesenchymal cells.

Glucose concentrations in blood are used to diagnose diabetes mellitus (DM). High blood glucose can damage blood vessels in the kidneys. However, the exact cause is unknown; the incorrect control of blood sugar and blood pressure is known to increase the risk of kidney damage [6]. High levels of glucose can be found in urine when it is excreted from the blood and result in glycosuria. Glycosuria is positive in patients with normal renal function when its serum concentration is above 10 mmol/L, and this value is even higher in patients with diabetic nephropathy [7].

Type 2 diabetes and hypertension are common comorbidities. Hypertension is twice as frequent in patients with diabetes compared with those who do not have diabetes [8]. Hypertension is the most important modifiable risk factor for all causes of morbidity and mortality worldwide and is associated with an increased risk of cardiovascular disease [9]. The nephrons are the functional units of the kidney that filter the blood and are supplied with a dense network of blood vessels, and high volumes of blood flow through them. With time, high blood pressure can cause arteries around the kidneys to constrict and narrow, and these damaged arteries are not able to deliver enough blood to the kidney tissue [6].

According to data from the International Diabetes Federation, the global prevalence of DM in adults was estimated at 8.8% in 2015 and might increase to 10.4% in 2040. DM can lead to various health complications, including cardiovascular diseases, renal dysfunction, vision problems, and even amputation [10]. Therefore, primary prevention is extremely important to reduce DM prevalence and its consequences. HBP is an important and well-established modifiable risk factor for cardiovascular, peripheral arterial, and renal diseases [8]. Although HBP is common in patients with type 2 DM, its role in the development of DM is uncertain. Several longitudinal studies have shown that individuals with HBP or even pre-HBP have a higher risk of developing DM than normotensive individuals [11].

Raman spectroscopy is an optical spectroscopic technique used to determine the vibrational modes of molecules based on the inelastic scattering process. Raman spectroscopy can be used to identify biochemical samples in vivo, non-invasively, and in real time. Moreover, it can be used in vitro to examine the biochemical compounds present in biological fluids, without using chemicals and reagents. The benefits of Raman spectroscopy, when compared with traditional biochemical techniques, rely on the possibilities of detecting selected biomolecules without the need for reagents (reduced costs), detecting and quantifying several biochemicals at a time due to the multivariate nature of a single Raman signal (rapidness), which may reduce possible analytical errors [12], since the molecules associated with some diseases have very specific spectral properties and are easily detected through Raman scattering [13].

Raman spectroscopy has been used for serum analysis and diagnosis of different diseases such as endometriosis, Huntington’s disease, COVID-19, and cirrhosis [14,15,16,17]. The application of Raman spectroscopy for urine analysis has been previously studied to assist in early diagnosis by identifying biomarkers of nutritional supplementation, urinary tract infections, and other diseases, including chronic kidney disease and bladder cancer [18,19,20,21,22,23,24], the greatest advantage of Raman spectroscopy being the rapid analysis of individual samples. In short, Raman spectroscopy has been used to detect and quantify the main components in urine such as urea, creatinine, glucose, and phosphate [18,19,21,22]. Phosphate is the most abundant intracellular anion within the body and is an important component that is present in multiple physiological processes that affect different organ systems [25]. Elevated serum phosphate levels are generally found in patients with moderate to severe chronic kidney disease [26].

In the present study, we evaluated the main biomarkers present in the urine of healthy volunteers (control-CT group), as well as diabetic (DM) and hypertensive blood pressure (HBP) patients (DM&HBP group) without impaired renal functions, to compare the Raman spectroscopy with the conventional biochemistry method (spectrophotometry being the gold standard) used for classification and diagnosis, since these patients are at risk of developing kidney disease. We performed exploratory analysis by principal component analysis (PCA) to describe the spectral differences between the groups and to correlate these differences with the changes in urine composition due to DM&HBP. A multivariate model based on partial least squares (PLS) regression was developed to predict the concentrations of the biomarkers compared to the standard method and to classify the spectra into the clinical groups studied. An innovation of the present study is the evaluation of five biomarkers in the urine: urea, creatinine, glucose, phosphate, and total protein; previous studies only evaluated urea, creatinine, and glucose [18,19]. De Souza et al. [18] and Bispo et al. [19] employed a classification model based on the quadratic discriminant analysis (QDA) applied to the Raman/PCA to classify spectra in one of the four groups based on clinical observations (control, DM + HBP without complications, DM + HBP with complications, and dialysis). In the proposed model, the classification accuracy obtained for the DM&HBP group (without complications) was 32%. In order to improve the classification obtained in these previous studies, considering that in most cases these patients with DM&HBP are asymptomatic, we employed PLS discriminant analysis applied to Raman spectra in urine to classify subjects with DM&HBP from healthy (control) ones. In addition, another innovative feature of this study was to perform classification using the concentrations of the biomarkers from a conventional spectrophotometry assay and the concentrations estimated by the PLS model applied to the Raman spectra used for diagnosis (populational screening).

## 2. Materials and Methods

**Figure 1:**
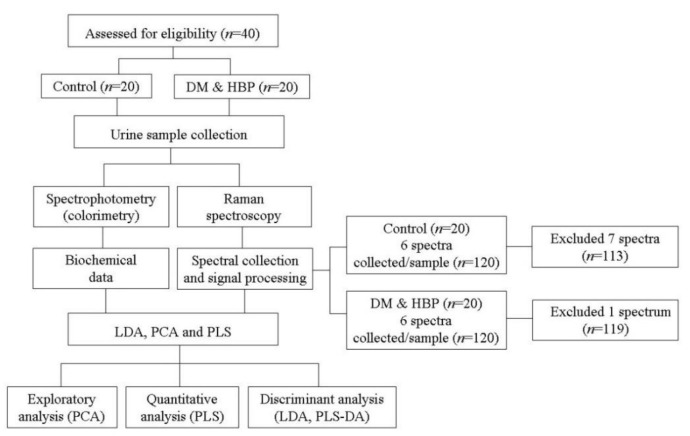
Diagram of the number of eligible samples assessed, the experimental setup, and data processing/analysis.

Figure 1 shows the diagram of the experimental setup, the number of eligible samples assessed, the total spectra included in and excluded from the study, and the data processing and analysis employed as detailed in the following sub-sections.

### 2.1. Urine Sample Collection and Biochemical Chemometric Assay

This study was approved by the Research Ethics Committee of Universidade Anhembi Morumbi (No. 2.717.746-C.A.A.E. 91318518.9.0000.5492), in accordance with the national guidelines and regulatory standards for research with human beings (Resolution No. 466/2012). A total of 40 patients (24 women and 16 men; age mean ± SD: 50 ± 16 years) were recruited and separated as follows: 20 healthy volunteers (normoglycemic and normotensive) in a control group (CT) and 20 individuals in a group of diabetics and hypertensive patients (DM&HBP) who did not have clinical and biochemical indicators of renal damage (Figure 1). The same procedure was used to collect the urine samples from the CT and the DM&HBP groups. Anamnesis was performed in both groups, and urine samples were collected in a sterile flask. Volunteers and patients were instructed to collect midstream (Type 1) urine during spontaneous urination, with prior hygiene of the genitals; that is, the first stream should be discarded, and the next midstream collected.

Samples of urine from the CT and DM&HBP groups were obtained from a health care unit of the Municipal Health Department in the city of Santarém, PA, Brazil. The subjects from CT and DM&HBP groups were fasting at the moment of sample collection, and the urine samples were placed in 2.0 mL cryogenic tubes and kept at −80 °C until spectral analysis. The transport of urine samples was carried out in an appropriate thermal box. The inclusion and exclusion criteria were the same for both groups. Subjects with a GFR within the reference values according to age and gender were included. Subjects who were presenting symptoms or clinical manifestations of urinary infection, who were not fasting before collection, or who reported symptoms of cardiovascular instability such as angina, palpitations, and hemodynamic decompensation were excluded.

After collection, the urine samples were submitted to biochemical chemometric analysis using an automated analytical spectrophotometric equipment (model Atellica CH 930 Analyzer, Siemens Healthcare Diagnósticos S.A., São Paulo, SP, Brazil, Table S1), and the values of urea, creatinine, glucose, and phosphate were obtained with specific kits (Atellica-ref. 11097593 for urea, ref. 11097533 for creatinine, ref. 11097592 for glucose and ref. 11097611 for phosphate). The measurement of total protein was conducted with a commercial kit (model Sensiprot, Labtest Diagnóstica S.A., Lagoa Santa, MG, Brazil) and the absorbance of the endpoint reaction was considered at 600 nm. Table S2 presents the calibration routine for the standard curves of the spectrophotometry assay and the linearity of the curves. The spectrophotometry experiments were conducted at a temperature of 20 °C (±1 °C) and a relative humidity of 60% (±10%), while Raman experiments were conducted at a temperature of 22 °C (±1 °C), and a relative humidity of 60% (±10%).

According to the instructions present in the analytical spectrophotometer kits, some urine compounds such as hemoglobin, conjugated and unconjugated bilirubin, and lipemia can interfere with the biochemical analysis, depending on the compound assayed. Interfering substances, tested according to the EP07-A2 guideline [27] and specific guidelines of the kit manufacturer (Atellica), are expected to have ≤10% interference in the assays. The total protein kit used has an interference of 5% in the assay.

Statistical analysis was performed to compare the concentrations of the biomarkers in the CT and DM&HBP groups using a Student’s t-test (*p* < 0.05, Instat software v. 3.05, GraphPad Software Inc., San Diego, CA, USA) preceded by a normality test (Kolmogorov–Smirnov, *p* < 0.1).

### 2.2. Raman Spectroscopy

Spectra collection was performed in a dispersive Raman spectrometer (model Dimension P-1, Lambda Solutions Inc., Waltham, MA, USA). The equipment uses an 830 nm multimode diode laser with an output power of about 400 mW. A fiber optic cable (Raman probe) (model Vector Probe, Lambda Solutions Inc.) was used to provide radiation to the sample and collect the signal; the use of a Raman probe allows repeatable excitation and signal collection geometry. The measured laser power at the probe tip was 350 mW with a beam waist of about 170 μm. The Raman experiments were conducted at a temperature of 23 °C (±1 °C) and a relative humidity of 60% (±10%). Urine samples were placed in an aluminum sample holder, approximately 80 μL in volume, with 4 mm diameter holes. The Raman probe was placed at 10 mm from the sample holder. The Raman signal was collected with a 5 s integration time. Six spectra were randomly collected from each sample, totaling 240 spectra for the 40 samples (*n* = 20 for each group), and a total of 8 spectra were excluded due to the low signal-to-noise ratio (SNR < 10). Figure 1 shows the number of samples eligible and the number of spectra used in the study.

The Raman spectra were then pre-processed in the following order: manual removal of cosmic rays spikes, automatic removal of the fluorescence baseline signal by fitting and subtracting a fifth-order polynomial, and automatic normalizing by the area of the water band at 1660 cm^−1^. This pre-processing was performed in a laboratory-made routine using Matlab software (version 7.4.0, The Mathworks Inc., Natick, MA, USA). The normalized mean Raman spectra of urine from the groups studied were plotted and compared.

### 2.3. Data Processing and Analysis—Exploratory Analysis and Linear Regression Models for Quantification and Discrimination

Figure 1 also shows the diagram of data processing and analysis. PCA was employed for exploratory analysis to identify the biochemical differences between the groups revealed by the Raman bands. Principal component scores (Scores), which are projections of the dataset onto the new principal components axes (the axes that maximize the variance in the data) [28], and the principal component coefficients (loadings or PCs), which are the projections (the intensities) of the data onto these new axes [28], were extracted and plotted. The Scores resemble the Raman spectra, with positive and negative peaks in the positions of the compounds present in the urine, and, together with the PCs, can show specific chemical information and be interpreted as differences in the biochemical composition of the groups [29]. Therefore, the spectral features presented in the Scores were correlated to the compounds found in the urine samples, and the PCs were used to identify where these differences occur. PCA calculation was performed using the Matlab software and the routine princomp.m.

The Student’s *t*-test (*p* < 0.05, Instat software v.3.05) preceded by a normality test (Kolmogorov–Smirnov, *p* < 0.1) was applied to the PCs to identify which one presented a difference in the CT and DM&HBP groups related to the biochemical information presented in the Scores.

A spectral model based on the PLS regression technique was applied to the Raman spectra of urine to estimate the concentrations of urea, creatinine, glucose, phosphate, and total protein using the spectrophotometric assay (gold standard) as inputs. The model considered the spectral information from the Raman signal as independent variables (*x* data) and the concentrations determined by conventional biochemical analysis (spectrophotometry) as dependent variables (*y* data) or the real concentration. Leave-one-out cross-validation was used, where *n*-1 samples were used in the PLS regression model, and the concentrations of a left-out sample were predicted using a certain number of latent variables (LVs). The model’s performance was evaluated by the root means square error of cross-validation (RMSEcv) and Pearson’s correlation coefficient (*r*), where the best model is the one that gives the minimum RMSEcv and maximum *r* as LVs are added to the model.

Discriminant models based on linear discriminant analysis (LDA) and PLS regression (PLS-DA) were used to classify the subjects into CT and DM&HBP groups. In all cases, the dependent variables (y data) were group classes or categories, “1” being samples within the reference values (CT) of the compounds and “2” being the samples out of the reference values (DM&HBP). The independent variables (*x* data) considered in each model were (a) the concentrations of creatinine, urea, glucose, phosphate, and total protein obtained by spectrophotometric analysis, modeled by LDA, (b) the concentrations of the compounds assayed by Raman spectroscopy, also modeled by LDA, and (c) the entire Raman spectra dataset, modeled by PLS-DA. In PLS-DA models, the performance is commonly evaluated by the overall accuracy (the number of correct classifications divided by the number of cases) [30], where the best model is the one that gives the highest accuracy as LVs are added to the model.

The Chemoface software [31] was used to perform PCA, PLS, and PLS-DA. The steps to perform PCA, PLS regression, and PLS-DA can be found elsewhere, e.g., in Chemoface—User Guide (http://www.ufla.br/chemoface) (accessed on 18 August 2022).

## 3. Results and Discussion

### 3.1. Raman Spectra

Table 1 shows the mean values of the concentrations of urea, creatinine, glucose, phosphate, and total protein in the urine of the two groups analyzed, CT and DM&HBP, assayed by spectrophotometry (gold standard). The comparative analysis between the CT and DM&HBP groups showed no statistically significant difference between groups in each of the five biomarkers analyzed (*p* ˃ 0.05).

In Table 1, it is clear that both groups presented urea, creatinine, and phosphate within the reference values (RVs), which was expected because the subjects included in the study did not have clinically diagnosed renal dysfunction. The concentration of glucose in the CT group was within the RVs. However, 25% of the volunteers in the DM&HBP group had glucose levels above the RVs. The high glucose levels in some individuals may be due to the lack of correct control drug therapy, where patients might not have been taking their prescribed medications correctly. Chronic hyperglycemia is associated with a significantly higher risk of developing diabetes-related microvascular and macrovascular complications. The early detection of DM through screening increases the likelihood of identifying asymptomatic individuals and enables adequate treatment to reduce diabetes and its complications [32]. The concentration of total protein was above the RVs in 45% of the subjects in the CT group, while it was above the recommended values in the urine of 35% of the subjects in the DM&HBP group. Proteinuria is a major marker of chronic kidney disease, and Raman spectroscopy has been proposed as an alternative method for diagnosing this health condition [33].

**Table 1:** Concentration of the main biomarkers of urine from the CT and DM&HBP groups determined using a spectrophotometry assay. Reference values from Wu [34] are converted to SI (mol/L).

Urinary Biomarker	Reference Values	CT Mean Concentration ± SD	CT Total Amount of Samples/No. of Samples Above RVs	DM&HBP Mean Concentration ± SD	DM&HBP Total Amount of Samples/No. of Samples Above RVs
Urea	51.6–549 mmol/L (M) 46.9–580 mmol/L (W)	271 ± 89 mmol/L	20/0	249 ± 68 mmol/L	20/0
Creatinine	2.12–34.6 mmol/L (M) 1.4–28.9 mmol/L (W)	12.5 ± 5.5 mmol/L	20/0	9.3 ± 4.0 mmol/L	20/0
Glucose	<0.83 mmol/L	0.23 ± 0.04 mmol/L	20/0	17.3 ± 38.7 mmol/L	20/5
Phosphate	1.6–61 mmol/L (M) 2.3–48 mmol/L (W)	23.4 ± 14.1	20/0	17.2 ± 9.9	20/0
Total protein	1–15 mg/dL	14.7 ± 5.5 mg/dL	20/9	15.6 ± 7.2 mg/dL	20/7

RV: reference value; M: men; W: women.

**Figure 2:**
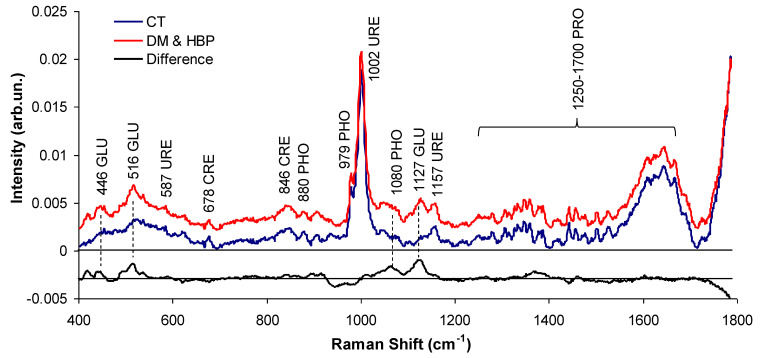
Mean Raman spectra of the CT and DM&HBP groups and difference spectrum (DM&HBP-CT). The assignments of the main peaks are detailed in the text. Spectra are offset for clarity. GLU: glucose; URE: urea; CRE: creatinine; PHO: phosphate; PRO: total protein.

Figure 2 shows the mean Raman spectra of the CT and DM&HBP groups in the 400–1800 cm^−1^ spectral range as well as the difference spectrum (DM&HBP-CT). The spectra are mostly dominated by Raman peaks corresponding to urea, creatinine, glucose (particularly for the DM&HBP group), phosphate, and proteins (also for the DM&HBP group). The urea peaks were at 516 (overlap with glucose), 587, 1002, and 1157 cm^−1^; the creatinine peaks were at 678, 846, and 910 cm^−1^ (overlap with glucose); the phosphate peaks were at 880, 979, and 1080 cm^−1^, the glucose peaks were at 446, 516 (overlap with urea), 910 (overlap with creatinine), 1080 (overlap with phosphate), and 1127 cm^−1^; protein peaks were between 1250–1700 cm^−1^. The overlap of some Raman bands (for instance, the 516 cm^−1^ peak) occurs due to similarities in the energy vibrations of carbon single bonding in such organic compounds, particularly, the C–C, C–N, and C–O modes (C–C–O bending for glucose [35] and N–C–N and N–C–O bending for urea [36]). The main difference between both groups was the presence of glucose peaks (446, 516, 1080, and 1127 cm^−1^). These Raman peaks have been previously described [19,21,32,35,36,37,38,39].

### 3.2. Exploratory Analysis

**Figure 3:**
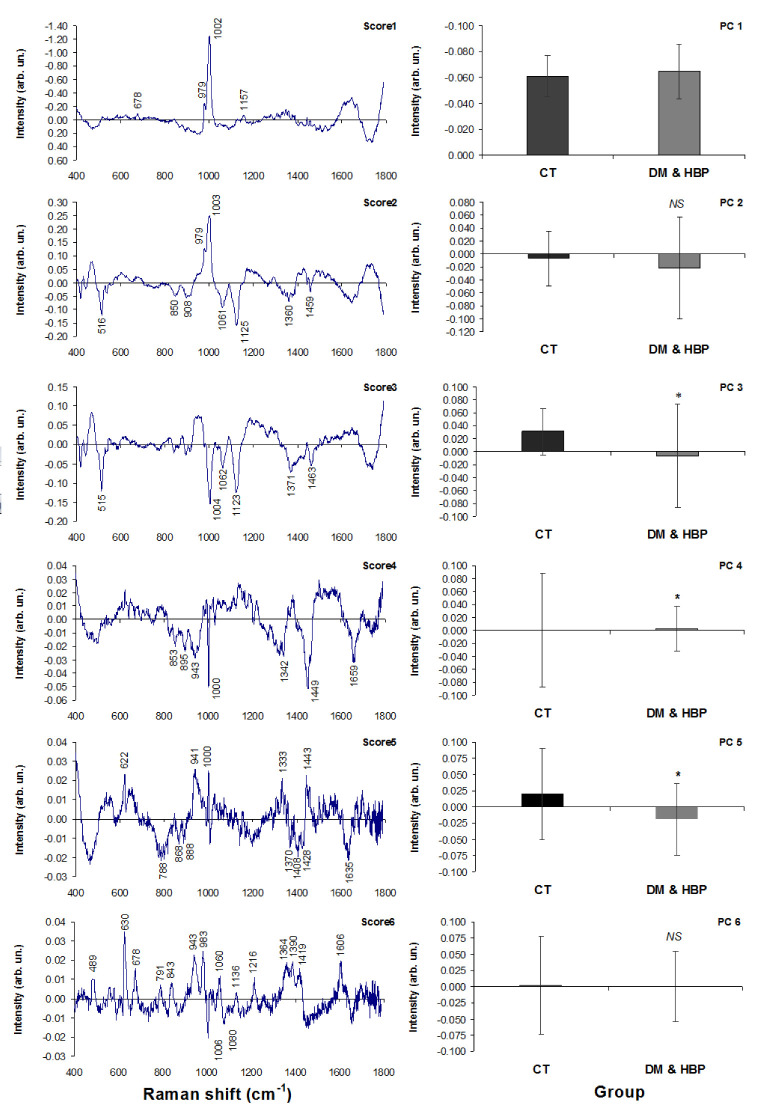
Plot of the first six principal component variables (Scores and PCs) extracted from the Raman dataset. The biochemical assignments seen in the Scores were based on the positions of the Raman bands compared to the literature. PC3, PC4, and PC5 show statistically significant differences between the groups (*t*-test, *p* < 0.05 *).

Exploratory analysis using PCA was used to reveal differences in the Raman features of the CT and DM&HBP groups. High spectral variations were found to be in the first six principal components (>98% of spectral variance), and the six principal component variables (PCs and Scores) are plotted in Figure 3. Score1 shows the general spectral characteristics of compounds found in urine: creatinine (678 cm^−1^), phosphate (979 cm^−1^), and urea (1002 and 1157 cm^−1^), PC1 shows that there is no statistically significant difference between the groups (*p* > 0.05). Score2 shows positive and negative characteristic spectral features, the positive ones being assigned to phosphate (979 cm^−1^) and urea (1003 cm^−1^) and the negative ones being assigned to glucose (516, 850, 908, 1061, 1125, 1360, and 1459 cm^−1^). PC2 shows that there is no statistically significant difference between the groups (*p* > 0.05) despite the presence of higher glucose features in the DM&HBP group (negative PC2 and negative glucose features in Score2), suggesting that some subjects may not have a proper glucose control. Score3 shows negative spectral features assigned to glucose (515, 1062, 1123, 1371, and 1463 cm^−1^) and urea (1004 cm^−1^). PC3 shows that there is a statistically significant difference between the groups (*p* < 0.05), where the CT group had lower amounts of glucose and urea (seen by the positive PC3 values and negative Score3 features), the glycosuria being a clinical characteristic expected in subjects of the DM&HBP group without proper control. Score4 evidences negative spectral features assigned to proteins (suggested features of albumin) at 853, 895, 943, 1000, 1342, 1449, and 1659 cm^−1^ [12]. PC4 shows a statistically significant difference between the groups (*p* < 0.05), with a lower intensity for the DM&HBP group (negative protein features and positive PC4); this may suggest proteinuria in some subjects. Score5 shows positive spectral characteristics of proteins (622, 941, 1000, and 1443 cm^−1^) and negative features of proteins (788, 868, 888, 1370, 1408, 1428, and 1635 cm^−1^). PC5 shows a statistically significant difference between the groups (*p* < 0.05), and the negative features appearing in the DM&HBP group (negative PC5) suggest that proteinuria is occurring in this group. Score6 shows positive spectral features of proteins/amino acids (489, 630, 678, 791, 843, 943, 983, 1060, 1136, 1216, 1364, 1390, 1419, and 1606 cm^−1^) and a negative feature assigned to amino acids (1006 cm^−1^). PC6 shows no statistically significant difference between the groups (*p* > 0.05). The presence of proteins and amino acid features in Score6 without a significant difference between the groups suggests a complex composition of urine independently on the group.

**Figure 4:**
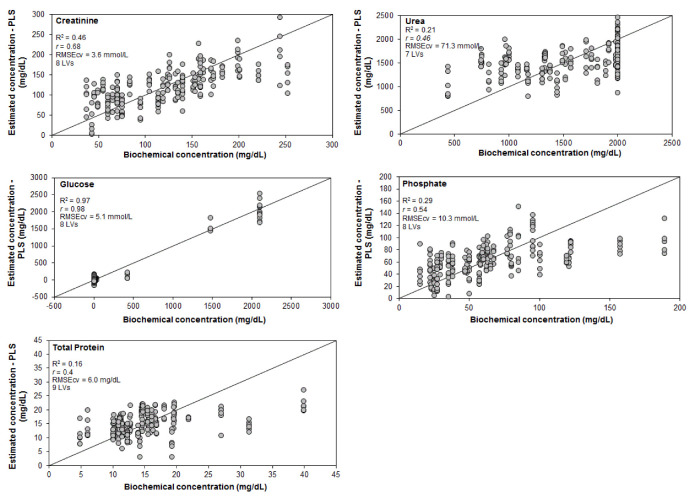
Correlation between the estimated concentrations of urea, creatinine, glucose, phosphate, and total protein in urine determined using spectral models based on PLS regression with leave-one-out cross-validation versus the concentrations determined by the spectrophotometry.

Figure 4 presents correlations between the real and estimated concentrations of the different biomarkers. The data revealed that creatinine and glucose were the biomarkers that presented the highest *r* between the two techniques analyzed (*r* = 0.68 and 0.98, respectively), both with 8 LVs, and RMSEcv = 3.6 and 5.1 mmol/L, respectively.

Table 2 presents the results of the discrimination model based on LDA to classify subjects into CT and DM&HBP groups using the concentrations of creatinine, urea, glucose, phosphate, and total protein obtained by spectrophotometric analysis. The LDA classification using the concentration values predicted by PLS and the PLS-DA classification based on the Raman spectra dataset considering 7 LVs, where all spectral information is considered, are presented. The highest accuracy of 81.5% was obtained by the PLS-DA model using the entire Raman spectra dataset, with a sensitivity of 81.5% and a specificity of 81.4%, overcoming the accuracy of 60.0% of the LDA using the concentrations of the spectrophotometric analysis.

The recruitment of volunteers followed the same criteria for both groups. In the CT group. Although the volunteers had proteinuria, the GFR remained within the normal range (inclusion criteria), thus indicating that the individuals did not have renal damage. Healthy individuals may present increased proteinuria after physical activity once CTs are no longer sedentary. Post-exercise proteinuria is commonly seen after physical activity [40]. According to the manufacturer’s technical instruction of the total protein kit, some components, such as inorganic phosphate, calcium, magnesium, creatinine, glucose, and urea, may interfere in the analysis, thus causing positive interferences of lower than 5%. On the other hand, uric acid, sodium citrate, sodium oxalate, and ascorbate cause negative interferences of lower than 5%.

McMurdy and Berger [20] reported the first use of Raman spectroscopy to measure creatinine concentrations in unaltered urine samples from a multi-patient population with a cross-validation error of 4.9 mg/dL (0.43 mmol/mL) compared with the error of the chemical method of 1.1 mg/dL (0.1 mmol/L). Bispo et al. [19] identified potential biomarkers in the urine of diabetic and hypertensive patients related to kidney disease. The quadratic discriminant analysis (QDA) revealed spectral characteristics of urea, creatinine, and glucose in the spectra of urine. A reduction in urea and creatinine concentrations was observed as the renal disease progressed, while urine and glucose increased as the prognosis worsened when compared with the control group. The QDA model presented an overall classification rate of 70%. Thus, the Raman spectroscopy associated with PCA and QDA was shown to be a valuable tool for identifying biomarkers in human urine, classifying urine samples according to disease status, and thus diagnosing renal diseases.

Saatkamp et al. [21] modeled the intensity of the Raman peaks of urea at 1006 cm^−1^ and of creatinine at 681 cm^−1^ versus the standard biochemical assay for predicting the concentrations of urea and creatinine in midstream (type 1) urine obtained from healthy patients using PLS, showing correlation coefficient values of *r* = 0.90 and *r* = 0.91 and prediction errors RMSEcv = 312 mg/dL (51.9 mmol/mL) and = 25.2 mg/dL (2.23 μmol/L) for urea and creatinine, respectively. These models were applied to quantify these metabolites in patients with and without renal damage due to DM&HBP, showing that urea and creatinine were significantly lower in subjects with DM&HBP, with 808 ± 866 mg/dL (135 ± 144 mmol/L) for urea and 28 ± 81 mg/dL (2.48 ± 7.2 mmol/L) for creatinine, compared to healthy subjects, with 1626 ± 686 mg/dL (271 ± 114 mmol/L) and 136 ± 63 mg/dL (12.0 ± 5.6 mmol/L) for urea and creatinine, respectively. Recently, Carswell, Robertson, and Senger [41] performed a preliminary study of fresh urine mixed with whole blood and performed evaluations using Raman spectroscopy and chemometrics (PCA and PLS); the amounts of macro- and microhematuria (blood in urine) were correlated with the amount of blood added to the urine, with correlation coefficients as high as 0.91 for high hematuria levels (0–20% *v*/*v*) using PLS. The prediction accuracy for detecting microhematuria (0–1% *v*/*v*) was 91% using PCA.

It is important to emphasize that, in practice, the analysis of biomarkers using spectrophotometry is carried out individually, using different measures and reagent kits for each analysis, and the methodology varies according to the commercial kit used. As for the analysis using Raman spectroscopy, it is possible to identify and quantify different urine components in a single analysis. A recent study by Hu et al. [24] showed that biomarkers can be efficiently detected by Raman spectroscopy if the urine sample readings are performed in both supernatant and sediment fractions.

In the present study, the classification of the spectra into the CT and DM&HBP groups using the whole Raman spectra dataset (where all the spectral information is considered) was performed, and the accuracy of the classification was 81.5%. The patients included in the DM&HBP group did not present complications resulting from these two diseases; in most cases, they are asymptomatic. In general, such patients seek medical care or adhere to treatment when they already have symptoms or lesions in the target organs. As this group is a borderline kidney disease, clinical follow-up with a sensitive diagnosis is essential to reduce progression and damage to organs such as the kidney and the circulatory system. It should be noted that discrimination using the biomarkers commonly used in the conventional biochemical analysis showed an accuracy of only 60.0%, indicating the need for new analytical methods to be studied and inserted into the medical practice. In this way, divergent clinical and laboratory classification would be minimized, to ensure the most appropriate therapy for each case.

The results suggest that Raman spectroscopy may be a technique of choice for monitoring patients who may develop complications resulting from diabetes and hypertension. Raman spectroscopy could be a viable option to replace traditional methods in the monitoring of patients who may develop complications resulting from these afflictions, with the advantage that the quantitative analysis of the five variables can be obtained from a single sample, thus reducing the time of the exam. In the near future, a method based on Raman spectroscopy for quantifying urine components could become an alternative to the existing methods for urinalysis or even replace them. This benefit would encourage patients with altered results detected in a Raman assay to seek more specific exams with lower error rates.

## 4. Conclusions

The exploratory analysis by PCA applied to the Raman spectra of urine from control (CT) compared to diabetic and hypertensive (DM&HBP) subjects showed that there were qualitative differences in the biochemical composition of urine, with marked peaks in glucose and total protein. The PLS regression model was able to estimate the concentration of urea, creatinine, glucose, phosphate, and total protein; creatinine and glucose were the biomarkers that presented the best correlation coefficient (*r*) between the two techniques analyzed (*r* = 0.68 and *r* = 0.98, respectively), both with 8 LVs and an RMSecv of 3.6 and 5.1 mmol/L (41 and 92 mg/dL), respectively. Discriminant analysis by PLS-DA using the entire spectra dataset was able to differentiate the samples of the groups in the study, with an accuracy (81.5%) higher compared to the LDA model (60.0%) using the concentration values of the spectrometric analysis. In clinical practice, Raman spectroscopy may have advantages over traditional biochemical techniques, as it can be used for urinalysis; moreover, it is possible to obtain several assays from a single sample spectrum, opening a window for the prediction of clinical complications resulting from diabetes and hypertension that may lead to the development of kidney disease in patients without clinical signs of complications.

## Figures and Tables

**Table 2:** Confusion table with the results of the discriminant models used to classify subjects into CT and DM&HBP groups using LDA applied to the concentrations of the biomarkers obtained by spectrophotometric analysis and concentrations predicted by PLS regression. The classification by PLS-DA using the entire Raman spectra dataset is also presented.

Classification According to the Clinical Criteria	Classification Using Spectrophotometric Analysis	
	Control	DM&HBP
Control (*n* = 20)	13	7
DM&HBP (*n* = 20)	9	11
Sensitivity	55.0%	
Specificity	65.0%	
Accuracy	60.0%	
**Classification According to the Clinical Criteria**	**Classification Using the Concentration Values Predicted by PLS**	
	**Control**	**DM&HBP**
Control (*n* = 113)	85	28
DM&HBP (*n* = 119)	42	77
Sensitivity	64.7%	
Specificity	75.2%	
Accuracy	69.8%	
**Classification According to the Clinical Criteria**	**Classification Using Raman Spectra by PLS (7 LVs)**	
	**Control**	**DM&HBP**
Control (*n* = 113)	92	21
DM&HBP (*n* = 119)	22	97
Sensitivity	81.5%	
Specificity	81.4%	
Accuracy	81.5%	

## Data Availability

Samples of the compounds are available from the authors.

## Supplementary Materials

The following supporting information can be downloaded at: https://www.mdpi.com/article/10.3390/bioengineering9100500/s1. Table S1. Main technical specifications and details of the automated spectrophotometric equipment used (Atellica CH 930 Analyzer). Table S2. Analyte Limit of Detection (LOD) and linearity of chemistry assays in the urine according to the manufacturer of the kit.
