# Supplementary material for: Biochemical Analysis of Urine Samples from Diabetic and Hypertensive Patients without Renal Dysfunction Using Spectrophotometry and Raman Spectroscopy Techniques Aiming Classification and Diagnosis
Source: Bioengineering (Basel). 2022 Sep 24;9(10):500. doi: 10.3390/bioengineering9100500 (PMC9598339; doi:10.3390/bioengineering9100500)
Supplement: Supplementary file 1 [file bioengineering-09-00500-s001.zip › bioengineering-1898280-supplementary.pdf]

Supplementary Materials

Table S1. Main technical specifications and details of the automated spectrophotometric equipment used (Atellica CH 930 Analyzer).

**Main technical specifications**	**Details**
General description	Chemistry analyzer with electrolyte (IMT) and photometric testing capabilities
Sample volume per test	Photometric: 0.4 µL to 5.0 µL (varies by assay)<br>IMT: 25 µL produces results for sodium (Na^+), potassium (K^+), and chloride ($Cl^–$)
Reaction detection photometer	11 fixed wavelengths (340, 410, 451, 478, 505, 545, 571, 596, 658, 694 and 805 nm)
Reagent inventory management	Automatic tracking and notification of remaining tests, onboard stability and expiration, calibration, and storage conditions for each pack and well
Average reagent volume	10–100 µL per test, assay-dependent
Auto-calibration	Automatic assay-specific lot and pack calibration (when connected to Atellica sample handler)

More technical specifications are available in the manufacture's website (Siemens Healthcare GmbH), available at: https://www.siemens-healthineers.com/integrated-chemistry/systems/atellica-solution-analyzers#TECHNICAL_DETAILS

Table S2. Analyte Limit of Detection (LOD) and Linearity of chemistry assays in the urine according to the manufacturer of the commercial kit.

Analyte	Limit of Detection (LOD)	Linearity
Glucose	2 mg/dL (0.1 mmol/L)	4–700 mg/dL (0.22–38.85 mmol/L)
Phosphate	0.5 mg/dL (0.16 mmol/L)	4.0–100.0 mg/dL (1.29–32.30 mmol/L)
Creatinine	0.02 mg/dL (2 µmol/L)	1.00–245.00 mg/dL (88–21658 µmol/L)
Urea	4 mg/dL (1.4 mmol/L)	35–1000 mg/dL (12.5–357.0 mmol/L)
Total Protein	0.12 mg/dL (0.0012g/L)	100 mg/dL (5.6 mmol/L)

The concentration calculation was performed from a standard curve, taking into account the linearity of each analyte presents in the urine (Table S2). For calibrating chemistry assays (glucose, phosphate, creatinine and urea) by the spectrophotometry was used for calibration.
